# A High-Throughput Microfluidic Magnetic Separation (µFMS) Platform for Water Quality Monitoring

**DOI:** 10.3390/mi11010016

**Published:** 2019-12-22

**Authors:** Keisha Y. Castillo-Torres, Eric S. McLamore, David P. Arnold

**Affiliations:** 1 Interdisciplinary Microsystems Group, Department of Electrical and Computer Engineering; University of Florida, Gainesville, FL 32611, USA; keishact@ufl.edu; 2 Institute of Food and Agricultural Sciences, Department of Agricultural and Biological Engineering; University of Florida, Gainesville, FL 32611, USA; emclamore@ufl.edu

**Keywords:** high-throughput, magnetic isolation, magnetic separation, magnetic microdiscs, microfluidics, bacteria, *Escherichia coli*, water quality

## Abstract

The long-term aim of this work is to develop a biosensing system that rapidly detects bacterial targets of interest, such as *Escherichia coli*, in drinking and recreational water quality monitoring. For these applications, a standard sample size is 100 mL, which is quite large for magnetic separation microfluidic analysis platforms that typically function with <20 µL/s throughput. Here, we report the use of 1.5-µm-diameter magnetic microdisc to selectively tag target bacteria, and a high-throughput microfluidic device that can potentially isolate the magnetically tagged bacteria from 100 mL water samples in less than 15 min. Simulations and experiments show ~90% capture efficiencies of magnetic particles at flow rates up to 120 µL/s. Also, the platform enables the magnetic microdiscs/bacteria conjugates to be directly imaged, providing a path for quantitative assay.

## 1. Introduction

Detection of bacteria indicative of fecal contamination is central to water quality monitoring for ensuring safe water for human contact and/or drinking. For instance, as reported in the National Outbreak Reporting System (NORS) from the Center for Disease Control and Prevention (CDC), between 2009 and 2017 there were a total of 33 outbreaks associated with exposure to fecal contaminated (*Escherichia*, *Enterococcus*, and *Streptococcus*) water (drinking, recreational, environmental, and undetermined), which resulted in 436 cases of illness, 65 hospitalizations, and 1 death in the United States [1,2]. Health effects such as gastrointestinal illnesses have been associated with exposure to fecal bacteria via drinking water [3,4]. Specifically, epidemiological studies by the U.S. Environmental Protection Agency (EPA) have shown strong correlations between illnesses and bacteria concentrations of *Enterococci* and generic *Escherichia coli* in fresh and marine waters [5]. Generic *E. coli* is used as the indicator of fecal contamination, although there are a host of pathogenic microorganisms that are responsible for the aforementioned health problems.

For safe human contact, it is recommended that recreational waters have a geometric mean (GM) below 35 CFU/100 mL for *Enterococci* and below 126 CFU/100 mL for *E. coli*, as specified by the Recreational Water Quality Criteria from the U.S. EPA [3]. This limit (126 CFU/100 mL) is identical to the hazard threshold set by the Food and Drug Administration for the irrigation of fresh produce according to the Food Safety Modernization Act (FSMA) [6]. In drinking water, the maximum level is set at 1 CFU/100 mL for *E. coli* [5], which is near the detection limit of many detection systems.

While there has been much work in bacteria detection for water and food samples [7,8], there are operational aspects that limit detection schemes for rapid, point of use applications [9]. Prominent limitations include long analysis times (typically 24 h including enrichment of bacteria), a need for complex lab tools/equipment, and/or the requirement for a highly skilled operator. The long-term aim of this work is to rapidly (<2 h) detect bacterial indicators of fecal contamination in 100 mL water samples pertinent to drinking water, recreational water, and food safety monitoring applications.

Microfluidic devices possess advantages in terms of their size, low-cost fabrication, and the possibility of parallel device operation [10]. Different microparticle/cell separation microfluidic technologies have been developed for large amount of particles/cells using acoustic, dielectric, thermal, or magnetic properties, among others as reviewed by Y. Shen, et. al, and T. Zhang, et al. in [11] and [12], respectively. Size-based microfluidic devices using deterministic lateral displacement (DLD) arrays for high-throughputs have been developed, using flow rates of up to 167 µL/s [13,14]. However, DLD structures usually consist of complex microfabrication process. Also, these DLD example devices possess an enrichment step and use multiple pumps [13] or have been tested to process up to 5 mL samples with a 91% targeted cell capture efficiency [14]. Microfluidic devices that make use of magnetic field gradients to enhance selectivity and increase throughput in cell separation and trapping applications have been developed [10,15,16,17,18,19,20]. 

Microfluidic magnetic separation technologies have aimed to reduce the total analysis time by avoiding long enrichment steps by isolating/concentrating magnetically-tagged bacteria using various magnetic field apparatuses [21]. Most magnetic separation biosensing systems for bacteria detection have been tested with sample volumes not larger than 10 mL, with limits of detection ranging 3.0 × 10^0^–1.5 × 10^9^ CFU/100 mL and analysis times ranging 0.35–2.5 h [21]. Previous works generally use magnetic beads having diameters 50–250 nm, where the beads comprise superparamagnetic iron oxide nanoparticles embedded in a polymer matrix (e.g., polystyrene). The net magnetic volume fraction of the bead is typically less than 15% vol. [17,22,23,24,25,26,27]. In contrast, the magnetic microdiscs used in this work are highly magnetic (88% vol), bacteria-sized discs (1.5 µm in diameter, 80 nm in thickness) and include a 5 nm layer of gold on each side, making them well-suited for magnetic separation of bacteria. In a previous work [21], aptamer-functionalized microdiscs were used to isolate *E. coli* at levels as low as 100 CFU/100 mL in less than 45 min. However, the isolation (magnetic trapping) step was performed using a bulky apparatus that required multiple successive passes through the device to achieve high capture efficiencies. Here, we present the use of a microfluidic device for faster sample filtering, convenient sample preparation, and ultimately better performance.

Main challenge of magnetic separation microfluidic devices is their typically small volume capacity. As summarized in Table 1, most of these magnetic separation microfluidic devices used sample volumes, ranging from a few μL to no more than 10 mL [16,17,19,20,28,29,30,31]. For these volumes, relatively low flow-rates, typically less than 20 µL/s, were sufficient to achieve results in short time [15,16,17,18,28,29,30,31]. For example, Zanini, et al. developed a microfluidic device with an integrated array of micromagnets with alternating polarities for the separation of magnetic nanoparticles, which resulted in > 94% particle capture efficiencies (with 0.25 µL/s flow rate) [28]. However, for water quality monitoring, there is need for processing much larger 100 mL samples in a short time period, which serves as motivation for this work.

In this work, we demonstrate the use of a much improved microfluidic magnetic separation (µFMS) device that offers flow rates of up to 120 µL/s with capture efficiencies of ~94%. This device is capable of analyzing 100 mL water samples in less than 15 min, a significant advancement towards rapid bacteria detection. Instead of integrating the magnets into the microfluidic platform as in [16,28,30], our device utilizes an external magnet array placed below the microfluidic platform, drastically simplifying the device fabrication and lowering the per-unit test cost. This proof-of-concept demonstrates isolation of microdisc/particle or microdisc/bacteria conjugates using a µFMS device, which uses a single filtration step protocol, providing an imaging-ready substrate for subsequent fluorescent microscopy.

**Figure 1:**
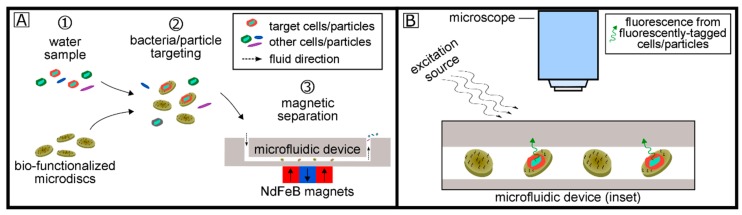
Overall concept on the biosensing system for bacterial target detection using bio-functionalized magnetic microdiscs and magnetic separation (**A**) and fluorescence imaging (**B**).

Figure 1 describes the overall concept of the biosensing system for bacteria detection. First, magnetic microdiscs bio-functionalized with specific capture probes (e.g., aptamers, antibodies, proteins) are used to separate bacterial/particle targets (e.g., *E. coli* or other target particles/cells). After co-incubation of these bio-functionalized microdiscs with a 100 mL water sample containing the target particle or cell, the μFMS device is used to isolate the microdisc/target conjugates in a localized area for imaging. Target cells can optionally be stained/labeled with a variety of fluorescent tags, and analysis is carried out using standard fluorescence microscopy.

## 2. Materials and Methods 

### 2.1. Fabrication of Magnetic Microdiscs

**Figure 2:**
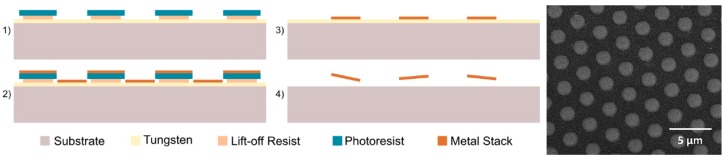
Microfabrication process of the magnetic microdiscs and scanning-electron microscopy (SEM) image of a densely-packed array of magnetic microdiscs on a substrate.

Magnetic microdiscs were fabricated using standard microfabrication techniques, as described in [21]. Briefly, a densely packed lithographically patterned array of circular holes (1.5 µm in diameter) were formed on a 100-mm-diameter silicon substrate predisposed with a 300-nm-thick sacrificial tungsten layer. A metal stack, consisting of 5 nm gold, 70 nm permalloy (Ni_80_Fe_20_), and then 5 nm gold again, was deposited by magnetron sputtering followed by an ultra-sonicated lift-off process using AZ400K developer (MicroChemicals, Ulm, Germany) diluted in water (1:4) to form the gold-coated permalloy magnetic microdisc array on the substrate. The total thickness of each magnetic microdisc was 80 nm. Finally, the microdiscs were released by dissolving the tungsten sacrificial layer using 30% hydrogen peroxide and washed/decanted three times using deionized (DI) water and a permanent magnet. Figure 2 shows the microfabrication process flow and a scanning-electron microscopy (SEM) image of the fabricated microdiscs before release from substrate. 

### 2.2. Bio-functionalization of Magnetic Microdiscs and Bacteria/Particle Targeting

The gold-coated microdiscs were bio-functionalized for selective binding to a bacterial/particle target using thiolated capture probes. In previous works, we targeted general coliforms and *E. coli* [21] using lectin- and aptamer-functionalized microdiscs, respectively. In this work, the same process of bio-functionalization for *E. coli*, using thiolated DNA aptamers (39 mer; MW = 11.8 kDa; K_D_ = 24.4 nM) synthesized by GeneLink (Hawthorne, NY, USA) was performed. The gold-coated magnetic microdiscs (~6 million discs) were suspended in 200–300 µL of capture probe (i.e., thiolated DNA aptamers) solution overnight at room temperature. The discs were then rinsed three times with deionized water using a permanent magnet to decant supernatant. Once magnetic microdiscs were functionalized, they were introduced into water samples containing *E. coli* for around 30 min at room temperature with occasional agitation. Then, samples were filtered using a simple magnet or a µFMS device followed by fluorescent staining, using the BacLight LIVE/DEAD kit, where 3 µL of SYTO9 per every 1 mL of sample was introduced. 

Additionally, for abiotic experimentation, we also tested and followed the same protocol described above using biotin-functionalized microdiscs to target avidin-coated polystyrene particles. Thiolated biotin (MW = 5 kDa, 10 mg/mL) purchased from NanoCS Inc. (Boston, MA, USA) was used to target avidin-coated particles. Avidin-coated fluorescent particles (yellow, 0.1 wt. %, 0.7–0.9 µm) were purchased from Spherotech, Inc (Lake Forest, IL, USA). 

### 2.3. Fabrication of µFMS Device

The microfluidic device was fabricated using a standard soft-lithography process [32]. Briefly, polydimethylsiloxane (PDMS) was prepared by mixing the base and curing agent solutions at a ratio of 10:1. The PDMS was then degassed using a vacuum chamber, deposited on a SU-8-silicon master mold (containing the desired microfluidic channel design), and degassed again. Next, the mold was cured in an oven at 65 °C for 4 h, treated with ultraviolet ozone (UVO) for 5 min to activate bonding between glass slide and PDMS, bonded, and placed in the oven for another 30 min to ensure chemical bonding between (PDMS and glass) surfaces. The master mold used here was fabricated using SU-8 and standard photolithography techniques on a silicon substrate. Each microfluidic device was designed with 8 microfluidic channels with dimensions of 2.1 mm × 45 mm and a height of 60 µm [33,34]. These 8 microchannels were originally connected to a single inlet and a single outlet. However, due to the high-throughput needs (100 mL samples, up to 120 µL/s), a total of 4 outlets were used to avoid pressure-induced delamination of the PDMS from the glass slide. 

Once the microfluidic (µF) component for the µFMS device was fabricated, the magnetic separation (MS) component was integrated considering the magnetic force acting on the magnetic particles. The magnetic force acting on a particle can be defined as:

(1) $${\vec{F}}_{m}=\left(\vec{m}\;\cdot \vec{\nabla \;}\right)\vec{B}$$

where $\vec{m}$ is the net magnetic moment of the particle and $\vec{B}$ is an applied magnetic flux density [35]. Similarly, the magnetic moment of a particle can be defined as,

(2) $$\vec{m}=vol\cdot \vec{M}$$

where vol is the volume of the particle and $\vec{M}$ is the particle magnetization [36].

**Figure 3:**
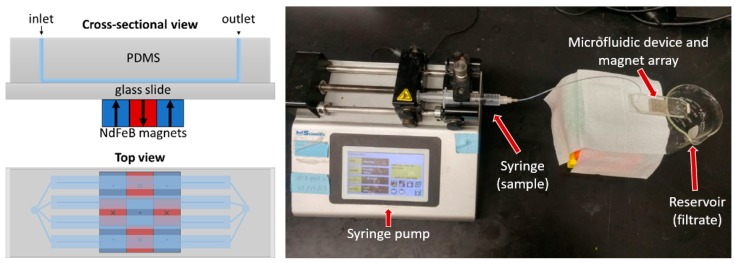
Magnetic separation microfluidic device diagram and experimental setup image (microfluidic device with magnets and syringe pump).

For the MS component, the magnetic assembly comprised a 3 × 3 array of neodymium (NdFeB) magnets (each a 6.35 mm × 6.35 mm × 6.35 mm cube) arranged with alternating polarizations in a chess-board pattern. The maximum field gradients (and hence magnetic forces acting on the magnetic discs) occurred at the boundaries between the magnets. The magnet array was attached to the glass slide of the microfluidic device using removable adhesive film. A syringe pump was used to control the flow rate of sample solutions into the microfluidic device during experiments. Figure 3 shows a schematic diagram of the magnetic separation microfluidic device and an image of the experimental setup.

### 2.4. µFMS Device Experiments

In this work, two main experiments were performed to evaluate capture efficiencies of the µFMS device using both microdiscs and iron-oxide nanoparticles (IONs): 1) separation of magnetic particles from 0.2 mL samples at various flow rates and 2) large-volume separation of magnetic particles from 50–100 mL samples. Considering the relationship between magnetic forces acting on particles, the magnetic moment of particles, and their volumes (Equations (1) and (2)), our microdiscs, larger in size and volume (diameter: 1.5 µm, thickness: 80 nm, volume: 1.24 × 10^−19^ m^3^) than typical individual IONs (diameter: 50–124 nm, volume: 6.54 × 10^−23^–99.8 × 10^−23^ m^3^), should experience higher magnetic forces resulting in better capture efficiencies during µFMS. 

First, experiments with microfabricated magnetic microdiscs were performed in both small- and large-volume samples (0.2 and 100 mL). Concentrations of magnetic microdiscs solutions tested were 6.5 µg/mL and 0.065 µg/mL for 0.2 and 100 mL samples, respectively. The concentration of 100 mL sample with microdiscs represented an immeasurable magnetic volume sample, however, it was limited due to microfabrication costs. Therefore, experiments with IONs were replicated for large-sample volumes with much higher concentrations of magnetic particles (12.5–100 µg/mL) in an effort to obtain a measurable magnetic volume sample for vibrating sample magnetometry (VSM) quantification (as will be described in the next sections), as well as to evaluate possible clogging of microchannels of the µFMS device. 

Small-volume sample solutions were prepared with 1) microfabricated microdiscs and 2) IONs (Fe_2_O_3_ nanopowder, < 50 nm particle size (BET)) purchased from Sigma-Aldrich (St. Louis, MO, USA). Then, five 0.2 mL samples of the disc solution, with a fixed concentration of ~6 million discs/mL, was filtered through the µFMS device at different flow rates: 5, 15, 30, 60, and 120 µL/s. While two 0.2 mL samples of the IONs solution, with a fixed concentration of ~3000 million particles/mL, were filtered through the µFMS device at two different flow rates: 5 and 120 µL/s.

Capture of microdiscs from large-volume samples (up to 100 mL) was assessed by filtering the samples with the µFMS device at a concentration of 0.065 µg/mL as a proof-of-concept. Then, capture of IONs was performed in 50 mL and 100 mL samples with concentrations of 100 µg/mL and 12.5 µg/mL, respectively. The importance of this experiment relied on our goal of isolating magnetic microdisc/bacteria conjugates from 100 mL samples in a short time period. Hence, these samples were filtered at a flow rate of 120 µL/s (up to 14 min filtering times) using a syringe pump with a 10 mL syringe (up to 10 times, for 100 mL samples). 

Finally, avidin-coated particles targeting and capture was assessed by adding the biotin-functionalized microdiscs to the 100 mL particle solution for 30 min at room temperature (protecting from light), followed by filtering using the µFMS device and a flow rate of 120 µL/s. Two 100 mL samples were prepared: 1) 3000 million particles and 2) 3000 particles. Both samples were exposed to the same amount of magnetic microdiscs (6 million). Similarly, a preliminary experiment was performed to isolate *E. coli* cells at a concentration of 100 CFU/mL from a 0.5 mL sample using aptamer-functionalized microdiscs at a flow rate of 120 µL/s.

The concentration of the magnetic particles in the stock (before filtration) and the filtrate (after filtration) of each of these samples (except *E. coli* sample) were quantified in order to estimate capture efficiencies, as will be described in the next section.

### 2.5. Estimation of Magnetic Particle Capture Efficiency 

The total magnetic moment of discs in solution was measured using a vibrating sample magnetometer (VSM), similar to a method described by C. M. Earhart, et al. [29]. The magnetic volume in the sample before and after filtration were estimated ($m=Vol\cdot M_{s}$), and the capture efficiency was calculated as:

(3) $$C.E.\%=\frac{Vol_{before\;filtration}-Vol_{after\;filtration}}{Vol_{before\;filtration}}\times 100$$

Sample preparation for the VSM measurements consisted of drying a pipetted droplet of known volume (10 to 500 µL) of the sample containing magnetic particles on a silicon substrate (~25 mm^2^). For example, in the case of the first experiment (0.2 mL samples), 0.1 mL filtrate droplets were dried on the substrate. On the other hand, for the second experiment (50 and 100 mL samples) 50 µL and 300 µL filtrate droplets were used for sample preparation. Supplementary Material Figures S1–S3 show example VSM data corresponding to samples before and after filtering with µFMS device for small-volume samples of microdiscs (0.2 mL), small-volume samples of IONs (0.2 mL samples), and large-volume samples of IONs (50 mL).

### 2.6. Multi-Physics Simulations

**Figure 4:**
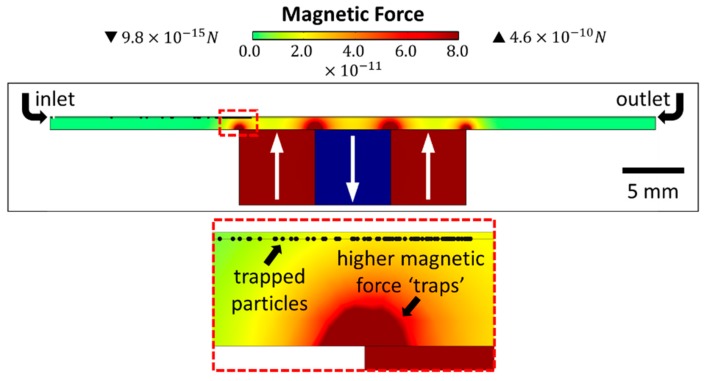
COMSOL geometry used for simulations of the µFMS device, isolating ‘microdisc’ particles with a *D_hd_* of 618 nm.

**Figure 5:**
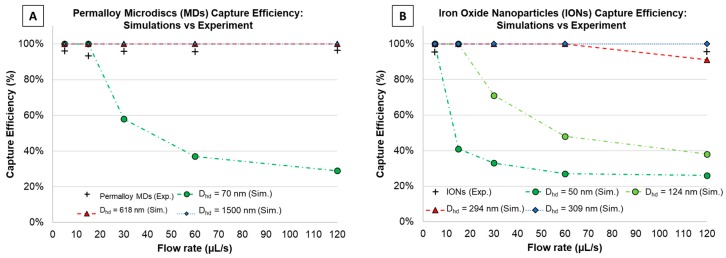
Capture efficiency study for (**A**) microdiscs and (**B**) iron-oxide nanoparticles (experiments and COMSOL simulations).

Magnetic capture of particles using 2D finite element modeling in COMSOL Multiphysics^®^ (COMSOL Inc., Burlington, MA, USA) was performed at different flow rates to match the experiment described before for confirmation. Here, the cross-sectional view presented in Figure 4 was designed, consisting of a single microfluidic channel (length: 45 mm, and height: 60 µm), 3 magnets (0.4 mm × 0.4 mm) with alternating polarizations, and a glass-slide spacing (1 mm) in between. Free triangular meshes were defined with minimum element sizes of 3 µm (for the microfluidic channel), 31.8 µm (for the glass slide), and 76.2 µm (for remaining structure). For this simulation, modules like ‘magnetic fields, no currents’, ‘laminar flow’, and ‘particle tracing for fluid flow’ were used. Here, two different kinds of particles were studied, IONs (Sigma-Aldrich < 50 nm) and our custom magnetic microdiscs (1.5 µm in diameter, 80 nm in thickness), which were simulated as spherical particles with three different hydrodynamic diameters (D_hd_): 70 nm (lower bound), 618 nm (equivalent magnetic volume of microdisc: 1.24 × 10^−19^ m^3^), and 1.5 µm (upper bound). In the case of the IONs, they were simulated with hydrodynamic diameters of 50 nm as defined by Sigma-Aldrich (< 50 nm) and 124 nm considering the hydrodynamic diameter measured in [37], as well as many other diameters accounting for particle aggregation. In Supplementary Material Figure S4, all *D_hd_* simulated for IONs are shown with their respective capture efficiencies. For the magnetic field, field gradient, and magnetic force calculations, Maxwell’s equations were solved within the ‘magnetic fields, no currents’ module. While the flow and particle velocity, the Navier-Stokes’ equations were solved within the ‘laminar flow’ and ‘particle tracing for fluid flow’ modules. Finally, the drag force acting on the particles and their trajectories were studied within the ‘particle tracing for fluid flow’ module. Figure 4 shows more details on the simulated geometry and Figure 5 summarizes the simulated and experimental capture efficiency results for the (A) microdiscs and (B) IONs. 

Simulations for each of the particles (IONs and microdiscs) were performed by changing the flow rates (*Q* = 5, 15, 30, 60, and 120 µL/s), to match later experimental results. However, considering that the flow rates set experimentally (using the syringe pump) were defined for a single inlet that then divides into 8 parallel channels, then in the simulations (for a single microfluidic channel) the flow rates were defined as: *Q_c_* = *Q*/8 ≈ 0.63, 1.9, 3.8, 7.5, and 15 µL/s. For simulations, 1000 particles were released in the inlet and simulated for 8 s with time steps of 0.01s. Finally, after each simulation, particles in the outlet were counted using the global evaluation (‘total number of particles in selection’ option), and capture efficiency was computed as follows:

(4) $$C.E.\%=\frac{\#Particles_{before\;filtration}\;-\;\#Particles_{after\;filtration}}{\#Particles_{before\;filtration}}\times 100$$

## 3. Results and Discussion

### 3.1. Magnetic Particle Capture Efficiency vs. Flow Rate 

Figure 5 shows the capture efficiency data vs. channel flow rate, for both experimental measurements (‘cross’ data points) and simulation results (dashed lines) for various hydrodynamic diameters. From the COMSOL simulations, Figure 5A predicts capture efficiencies of magnetic microdiscs (simulated as spherical particles) with *D_hd_* of 618 nm and 1.5 µm as 100%, while for the smaller diameter (70 nm) capture efficiencies decreased as the flow rates increased. Figure 5B shows how capture efficiencies of IONs in the µFMS device decreased with higher flow rates for *D_hd_* smaller than 124 nm, which experimentally was not matched. However, in order to match experiments, capture efficiency simulations were close to 100% for *D_hd_* of 294 nm or higher, which may serve as an indication of particle aggregation during experiments.

Experimental results for microdiscs (Figure 5A), showed capture efficiencies of 94.5 ± 1.8%, 93.3 ± 2.2%, 94.6 ± 1.8%, 95.0 ± 1.8%, and 95.4 ± 1.6% for 5, 15, 30, 60, and 120 µL/s flow rates, respectively. These results closely match the 100% COMSOL simulation results for discs (> 618 nm,). Similarly, results for IONs (Figure 5B), showed capture efficiencies of 94.7 ± 1.3% and 94.4 ± 1.1% for 5 and 120 µL/s flow rates, respectively, which closely matches COMSOL simulation results for particles with *D_hd_* > 294 nm. All percentages are represented as estimated mean with 95% confidence interval. Tables S1 and S2 in Supplementary Material summarize the estimated means, standard deviations, and 95% confidence interval values for experimental data.

### 3.2. Filtering Magnetic Nanoparticles from Large-Volume Samples

**Figure 6:**
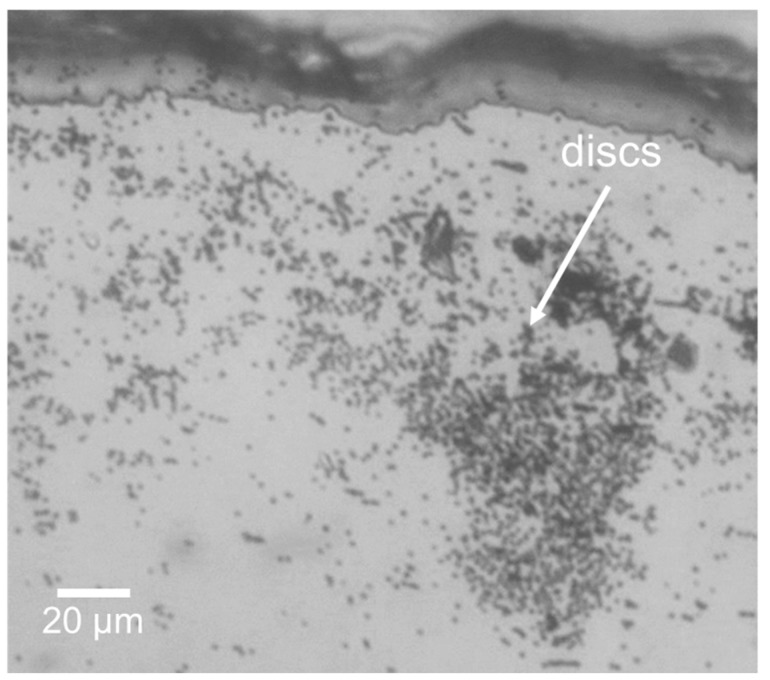
Example image of magnetic microdiscs captured from a 100 mL sample at 120 µL/s using the µFMS device.

**Figure 7:**
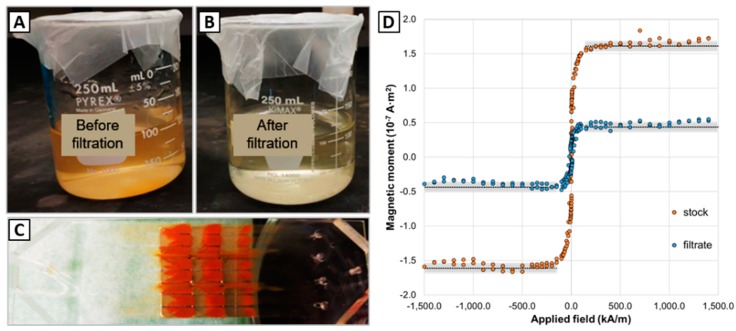
(**A**,**B**) 100 mL sample comparison: before filtration, after filtration, (**C**) IONs captured in the magnetic separation microfluidic device, and (**D**) vibrating sample magnetometer data corresponding to 300 µL dried droplet on a substrate from the 100 mL sample filtered using the µFMS device at 120 µL/s. In panel (**D**), black dashed lines represent the average value from the saturation magnetic moment measured and the gray bands represent ± one standard deviation.

The 100 mL sample containing ~6 million magnetic microdiscs was filtered at a flow rate of 120 µL/s using the µFMS device and imaged using an optical microscope. Figure 6 shows successful confirmation under the microscope of magnetic microdiscs trapped in the µFMS device. It is important to note that the concentration of the discs in solution was very low (0.065 µg/mL) compared to the solutions containing IONs (12.5–100 µg/mL) (Figure 7). Therefore, capture efficiency estimation (using the VSM) was not possible at this low concentration level.

From the large-volume samples experiments with IONs, the magnetic separation microfluidic device was capable of processing > 50 mL samples (Figure 7). The 50 mL sample (IONs concentration of 100 µg/mL) was filtered in ~7 min at a flow rate of 120 µL/s and the estimated capture efficiency was 70.0 ± 2.3%. Similarly, the 100 mL sample (IONs concentration 12.5 µg/mL, 100 mL) was filtered in ~15 min at a flow rate of 120 µL/s and the estimated capture efficiency was 72.2 ± 2.0%. Figure 7 show 100 mL sample experiment images of the solutions before filtering (A), after filtering (B), the µFMS device with all captured IONs (C), and the VSM data to estimate capture efficiency for 100 mL sample (D).

VSM data in Figure 7D shows the magnetic moment measured for the stock concentration (before µFMS filtration, orange data points) and filtrate (after µFMS filtration, blue data points) for the 100 mL sample. Additionally, the black dashed line represents the average saturation magnetic moment for each of the samples (1.52 × 10^−7^ A·m^2^ for the 300 µL from the stock sample, and 4.2 × 10^−8^ A·m^2^ for the 300 µL from the filtrate sample), which resulted in the 72.2 ± 2.0% capture efficiency.

These experimental results demonstrated that the µFMS device is capable of filtering > 50 mL samples at flow rates of 120 µL/s. Now, it can be observed that the capture efficiencies measured in these large-volume experiments (~72%) are lower than the ~93% reported for the experiments performed on 0.2 mL. However, it is important to consider that the 0.2 mL sample experiments consisted of a lower concentration (~6.47 µg/mL) of particles (microdiscs). Therefore, it is possible that this decreased capture efficiency was related to clogging of the µFMS device microchannels with such high concentrations of IONs in the 50 and 100 mL samples (100 and 12.5 µg/mL, respectively) (Figure 7C). This decreased capture efficiency might be related to the decreased free flowing cross-sectional area in the microchannels for the particles to move after clogging starts. This will cause an increase in the velocities experienced by the particles (inversely proportional to the cross-sectional area of the channel) and consequently increasing the drag forces acting on them, which may surpass the magnetic force.

### 3.3. Bacterial/Particle Target Isolation

**Figure 8:**
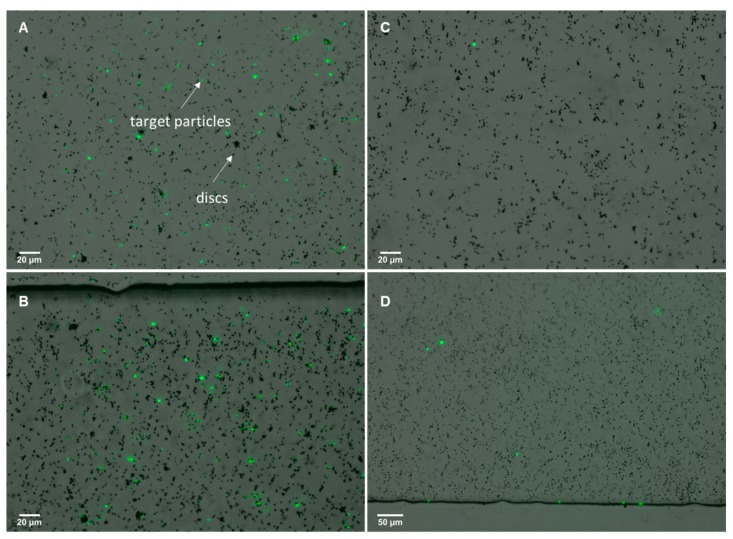
Isolation of fluorescent avidin coated (target) particles from 100 mL samples using biotin-functionalized magnetic microdiscs. (**A**,**B**) 100 mL sample containing 3000 million particles and (**C**,**D**) 100 mL sample containing 3000 particles.

Results on the isolation of particle targets were obtained for avidin-coated particles in 100 mL samples. Here, isolation of particles was successfully observed after filtration using the µFMS device and a flow rate of 120 µL/s. Two 100 mL samples with different particle concentrations were filtered using the µFMS device, one sample contained 3 × 10^−7^ particles/mL and the second sample contained 3000 particles/mL. Results show higher fluorescence in the sample with higher concentration of particles (Figure 8 A,B), when compared to sample with lower concentration of particles (Figure 8 C,D). Although the ‘target’ particle was not *E. coli* for this particular experiment results, proof-of-principle is similar. This study is important as it is based on a well documented/understood capture system (avidin/biotin). This material was by no means a control experiment, but provides strong insight into the feasibility of the proposed bacteria detection system.

**Figure 9:**
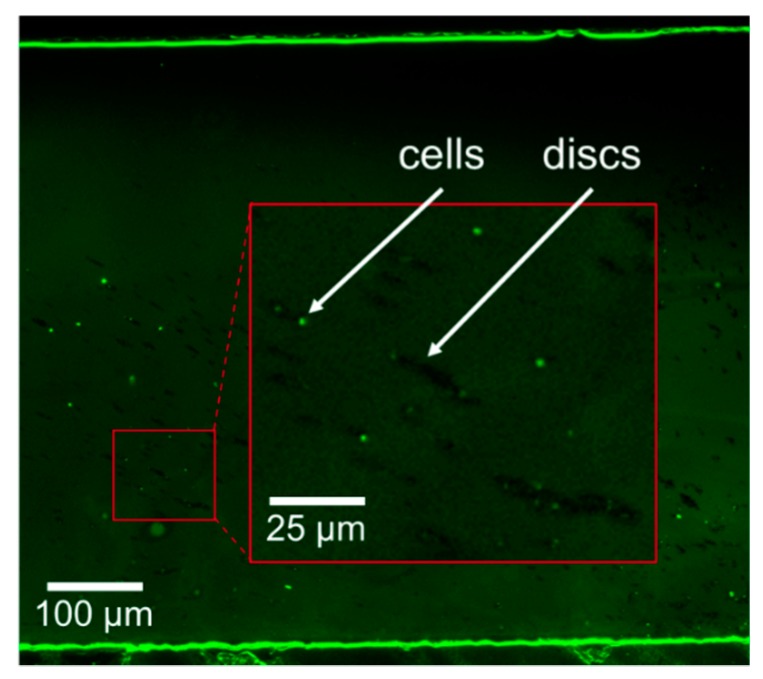
Example image of isolated aptamer-functionalized magnetic microdiscs/bacteria conjugates using the µFMS device and a flow rate of 120 µL/s.

Finally, preliminary results on the isolation of *E. coli* cells, from a 0.5 mL sample, using aptamer-functionalized microdiscs were obtained using the µFMS device and a flow rate of 120 µL/s. Confirmation of microdisc/bacteria conjugates was achieved via fluorescent microscopy. Figure 9 shows microdiscs and bacteria conjugates isolated in the microfluidic device. For this preliminary experiment bacteria concentration was 100 CFU/mL, while microdiscs concentration was also ~6 million discs/mL. 

## 4. Conclusions

In this work, a microfluidic magnetic separation (µFMS) device capable of filtering up to 100 mL samples in less than 15 min was developed. Also, capture efficiency studies in (0.2 mL samples) was > 90% at flow rates of up to 120 µL/s flow rates. Preliminary results have also shown promise towards 100 mL filtering using the same microfluidic device as well as isolating microdisc/bacteria conjugates, which at the same time results promising towards water quality monitoring tests. Future works will focus on the integration of the magnetic separation microfluidic device with a portable microscopy platform along with the image-processing algorithm for near-quantification of bacterial targets and microdiscs in field samples.

## Figures and Tables

**Table 1:** Summary of microfluidic magnetic separation recent works.

Lab-Prepared Sample Type (Target Particle/Cell)	Sample Volume (µL)	Flow Rate (µL/s)	Reference
Water (magnetic particles)	5	0.25	[28]
Blood *(E. coli)*	10	0.011	[17]
Water (*E. coli* and *Acinetobacter* sp.)	25	0.017	[16]
Water (*E. coli*)	nr *	0.833	[15]
Water (magnetic particles)	nr *	0.017	[18]
MACS (magnetic particles)	200	0.278	[29]
Buffer (Jurkat cells)	1000	0.333	[30]
Blood (*E. coli*)	2000	16.67	[31]
Blood (circulating tumor cells)	10,000	2.780	[19]
Blood (*Candida albicans* fungi)	10,000	5.556	[20]
Water (avidin-coated particles and *E. coli*)	up to 100,000	120	This work

* nr: not reported.

## Supplementary Materials

The following are available online at https://www.mdpi.com/2072-666X/11/1/16/s1. Figures S1–S3: VSM data for small-volume samples (0.2 mL) of microdiscs and small- and large-volume samples (0.2 and 50 mL) of IONs filtered at different flow rates using the µFMS device. Figure S4: Capture efficiency results from COMSOL for IONs with different hydrodynamic diameters. Table S1 and S2: Summarized capture efficiency data for microdiscs and IONs from 0.2 mL sample filtering.
