# Supplementary material for: A High-Throughput Microfluidic Magnetic Separation (µFMS) Platform for Water Quality Monitoring
Source: Micromachines (Basel). 2019 Dec 22;11(1):16. doi: 10.3390/mi11010016 (PMC7019623; doi:10.3390/mi11010016)
Supplement: Supplementary file 1 [file micromachines-11-00016-s001.pdf]

micromachines

MDPI

Supplementary Materials

A High-Throughput Microfluidic Magnetic Separation (µFMS) Platform for Water Quality Monitoring

Keisha Y. Castillo-Torres [1], Eric S. McLamore [2] and David P. Arnold [1,*]

[1] Interdisciplinary Microsystems Group, Department of Electrical and Computer Engineering; University of Florida, Gainesville, FL 32611, USA; keishact@ufl.edu
[2] Institute of Food and Agricultural Sciences, Department of Agricultural and Biological Engineering; University of Florida, Gainesville, FL 32611, USA; emclamore@ufl.edu
* Correspondence: darnold@ufl.edu

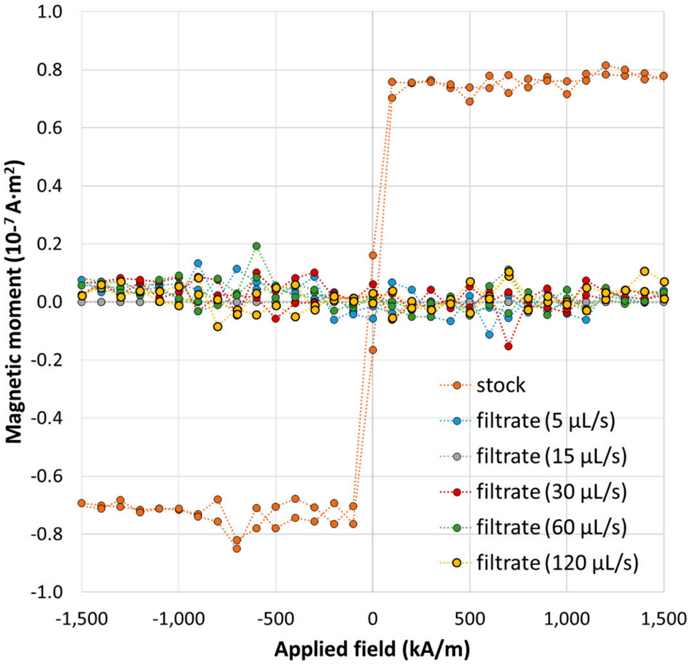

Figure S1. Vibrating sample magnetometer (VSM) data obtained (before and after filtration) from 0.2 mL samples filtered using the microfluidic magnetic separation (µFMS) device at different flow rates : 5 µL/s, 15 µL/s, 30 µL/s, 60 µL/s, and 120 µL/s. Table S1 summarizes the saturation magnetic moment for each sample, as well as the capture efficiency (%).

Table S1. Summary of Capture Efficiency (%) Calculations for Microdiscs.

Sample	Saturation Magnetic Moment (10^{-7} A·m²)			Capture Efficiency (%) (95% CI)
	Mean	Std. Dev.	Uncertainty (95% CI; N=56)	
**stock**	0.745	0.038	0.199	-
filtrate (5 µL/s)	0.041	0.029	0.008	94.5 ± 1.8
filtrate (15 µL/s)	0.050	0.036	0.010	93.3 ± 2.2
filtrate (30 µL/s)	0.040	0.031	0.008	94.6 ± 1.8
filtrate (60 µL/s)	0.037	0.033	0.009	95.0 ± 1.8
filtrate (120 µL/s)	0.034	0.026	0.007	95.4 ± 1.6

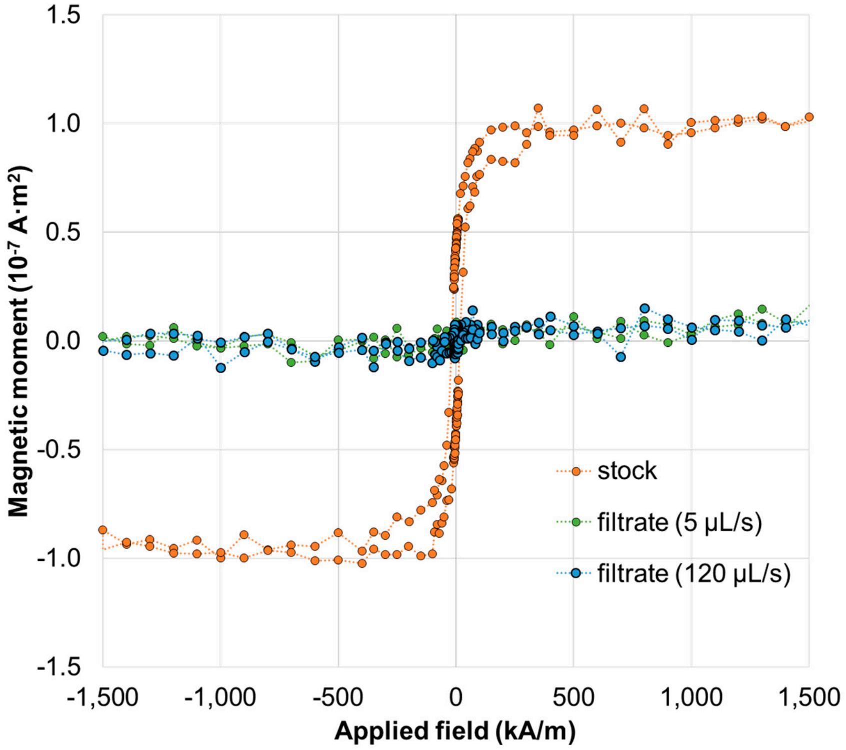

Figure S2. VSM data obtained (before and after filtration) from 0.2 mL IONs samples filtered using the microfluidic magnetic separation (µFMS) device at different flow rates: 5 µL/s and 120 µL/s. Table S2 summarizes the saturation magnetic moment for each sample, as well as the capture efficiency (%).

Table S2. Summary of Capture Efficiency (%) Calculations for IONs.

Sample	Saturation Magnetic Moment (10^{-7} A·m²)			Capture Efficiency (%)
	Mean	Std. Dev.	Uncertainty (95% CI; N=40)	
**stock**	0.972	0.046	0.015	-
filtrate (5 µL/s)	0.051	0.041	0.013	94.7 ± 1.3
filtrate (120 µL/s)	0.054	0.034	0.011	94.4 ± 1.1

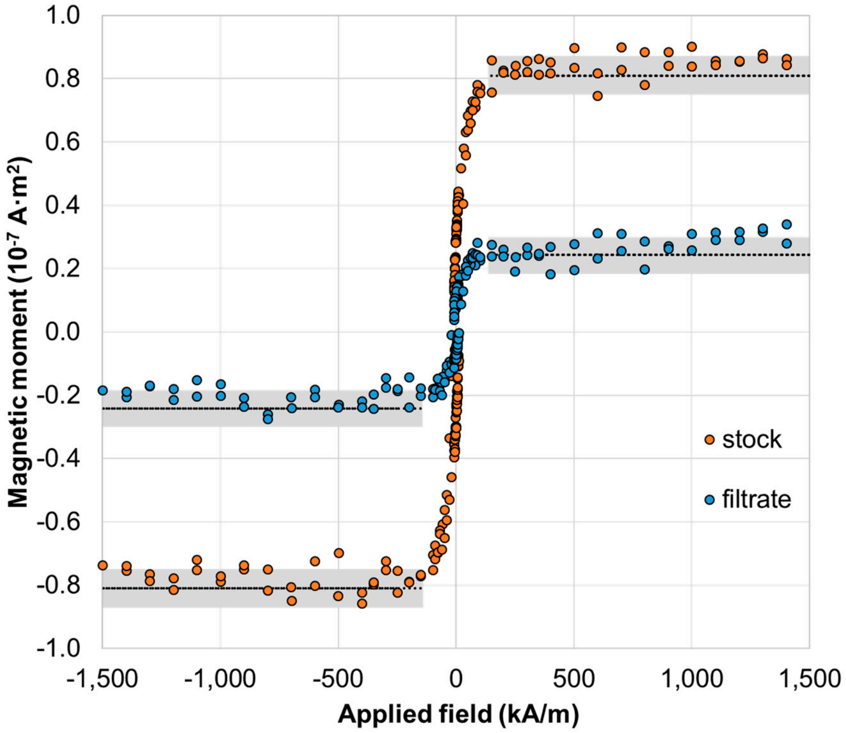

Figure S3A. VSM data obtained (before and after filtration) from 50 mL sample containing iron-oxide nanoparticles (IONs) at a concentration of 0.1 mg/mL filtered using the μFMS device at 120 μL/s. The black dashed line represents the average saturation magnetic moment for each of the samples (8.10 $\times 10^{-8}$ A·m^2 for the 20 μL from the stock sample, and 2.43 $\times 10^{-8}$ A·m^2 for the 20 μL from the filtrate sample), which resulted in the 70.0% capture efficiency.

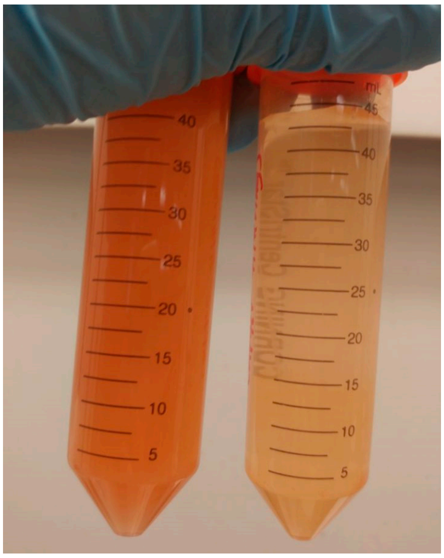

Figure S3B. Images of 50 mL sample (A) before and (B) after filtration using the μFMS device at 120 μL/s.

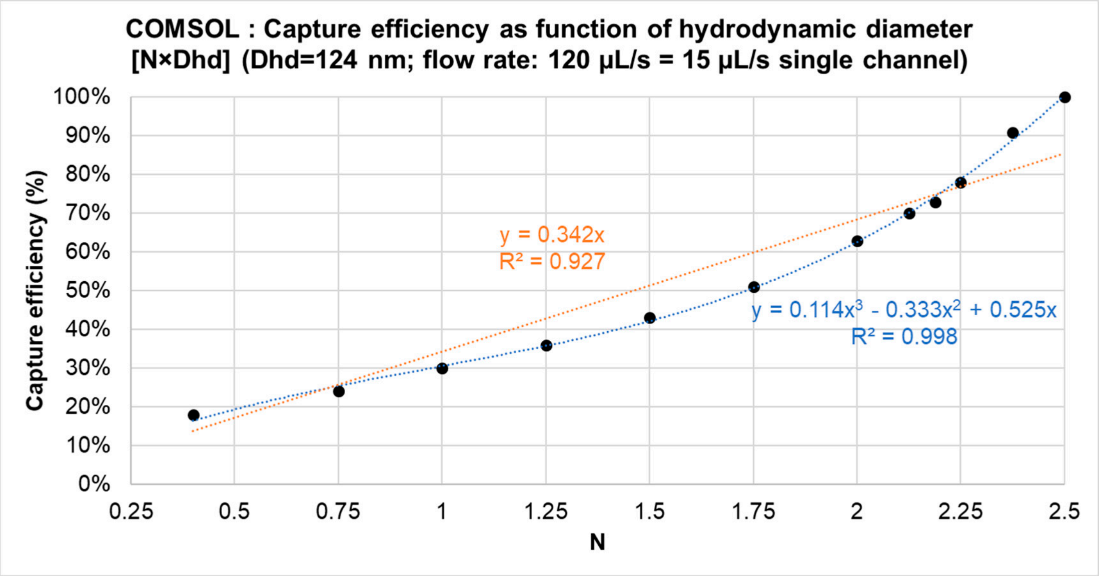

Figure S4. Plot of simulated capture efficiencies (COMSOL) as a function of change in hydrodynamic diameter of IONs, considering possible aggregation of particles. It is shown how the capture efficiency increases as the hydrodynamic/magnetic diameter increases.
